# A database of zooplankton abundance in the Atlantic sectors of the Southern and sub-Arctic Oceans

**DOI:** 10.1016/j.patter.2022.100554

**Published:** 2022-08-30

**Authors:** Peter Ward, Geraint A. Tarling, Petra ten Hoopen

**Affiliations:** 1 British Antarctic Survey, High Cross, Madingley Road, Cambridge CB3 0ET, UK

**Keywords:** zooplankton, Bongo net, Southern Ocean, Arctic Ocean, boreal, UK Polar Data Center

## Abstract

Scientific sampling of zooplankton in the Atlantic sector of the Southern Ocean has been undertaken since the 1920s, but few analyzed datasets are available to the research community. We provide a database of standardized data derived from samples collected by Bongo nets in this sector between 1996 and 2013, amounting to almost 94,000 individual records. The study region contains some of the highest levels of pelagic biomass in the Southern Ocean and is also undergoing rapid ocean warming and changing seasonality in sea-ice distribution. Data from a single expedition to the sub-Arctic where the same sampling methodology was used are also included. Atlantic water is an increasing influence in that region, as is the prevalence of boreal plankton taxa within Arctic plankton communities. These data will be of value in supporting studies assessing the impacts of climate change on the structure and function of polar pelagic systems.

## Highlights

• Plankton data from 15 recent scientific polar cruises are made available
• Analytical methodology, constraints, and assumptions are described in detail
• Analysis of similar data from 1920s to 30s shows no change in community distributions

## The bigger picture

• Polar oceans are undergoing significant change. As sea temperatures increase, so does the importance of understanding the factors underpinning the distribution and composition of plankton, its relationship to ocean physics, and its linkages in the food web. An understanding of species distributions and community structure provides a benchmark against which past and future change can be assessed.
• We wish to provide data for modelling how plankton communities respond to their changing environment, particularly temperature and food, and to predict possible future outcomes for communities and individuals.
• Societal impacts include a clearer understanding of how such changes may impact fisheries and carbon export, particularly in the Southern Ocean.

## Teaser

Zooplankton data collected during 15 oceanographic cruises to Polar regions have recently been made available through a global data portal. We describe how plankton were captured using bongo nets, analytical methodologies used, and constraints and assumptions made in producing standardized data. We describe how these data have been used for analyzing regional community distributions, biodiversity, and plankton growth and production. Historical comparisons show no changes in spatial distributions over the last 80 years, despite surface temperature having risen by ∼1°C.

## Introduction

Plankton support aquatic food webs providing food for higher trophic levels and commercially important fisheries. Phytoplankton are microscopic plants that sit at the base of aquatic food webs, absorbing nutrients and atmospheric CO_2_, which they fix in their tissues through photosynthesis. Zooplankton are microscopic aquatic animals that graze on phytoplankton and other microbes to meet their metabolic needs and facilitate growth and reproduction. Knowledge of the composition of plankton communities, their life cycles, and interactions with ocean physics, is key to understanding structure and function of the marine environment.

**Figure 1:**
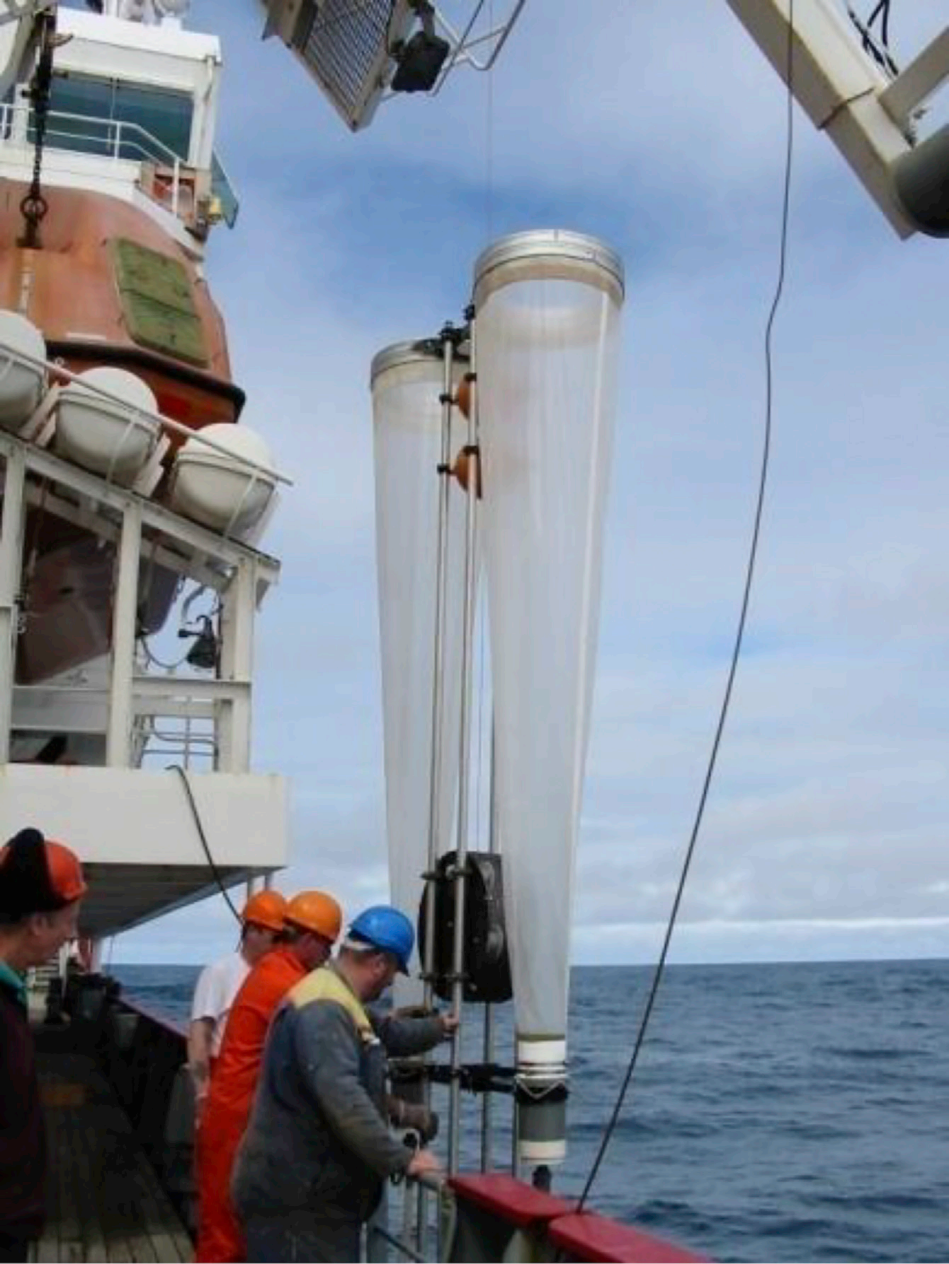
Motion-compensated Bongo net Springs providing the motion compensation are housed within the large circular cage between the two nets. The device contains solid cod-ends, below which are taps to release the sample into collecting buckets.

Historical interest in sampling plankton dates back to the early 19th century when, according to Fraser,^1^ Thompson used nets to capture crab and barnacle larvae in 1828. A few years later, Darwin sampled plankton with a net during the second voyage of the Beagle.^2^ At this time, research was largely opportunistic and curiosity driven using simple sampling techniques. Nearly a century later, as scientists sought to understand the geographical and seasonal distribution of plankton and their relation to environmental parameters in the ocean, different net systems proliferated, particularly from the 1950s onward.^3^ Simple vertically towed non opening/closing ring nets were commonly used in early studies and as such the Bongo nets (Figure 1) used in this study represent a development of these early attempts. Bongo nets have been widely used since the middle of the 20th century^4^ and consist of two plankton ring nets of relatively small mouth diameter mounted next to each other, each equipped with nets shaped like long funnels. Both nets terminate in separate cod-ends that collect the captured plankton.

Ten Hoopen et al. (2022, see the related paper^5^ in this issue of *Patterns*), describe the biological data publishing pipeline through which datasets describing plankton samples collected by Bongo nets in the Atlantic sector of the Southern Ocean and a single expedition to the sub-Arctic between 1996 and 2013 have been made globally accessible.^6^ In this paper we describe the methodologies used to collect and analyze these plankton samples, the constraints and assumptions in our analyses, and the studies that have so far resulted from these data that consider the ecology of mesozooplankton in the Southern Ocean.

## Results

### Data records

**Figure 2:**
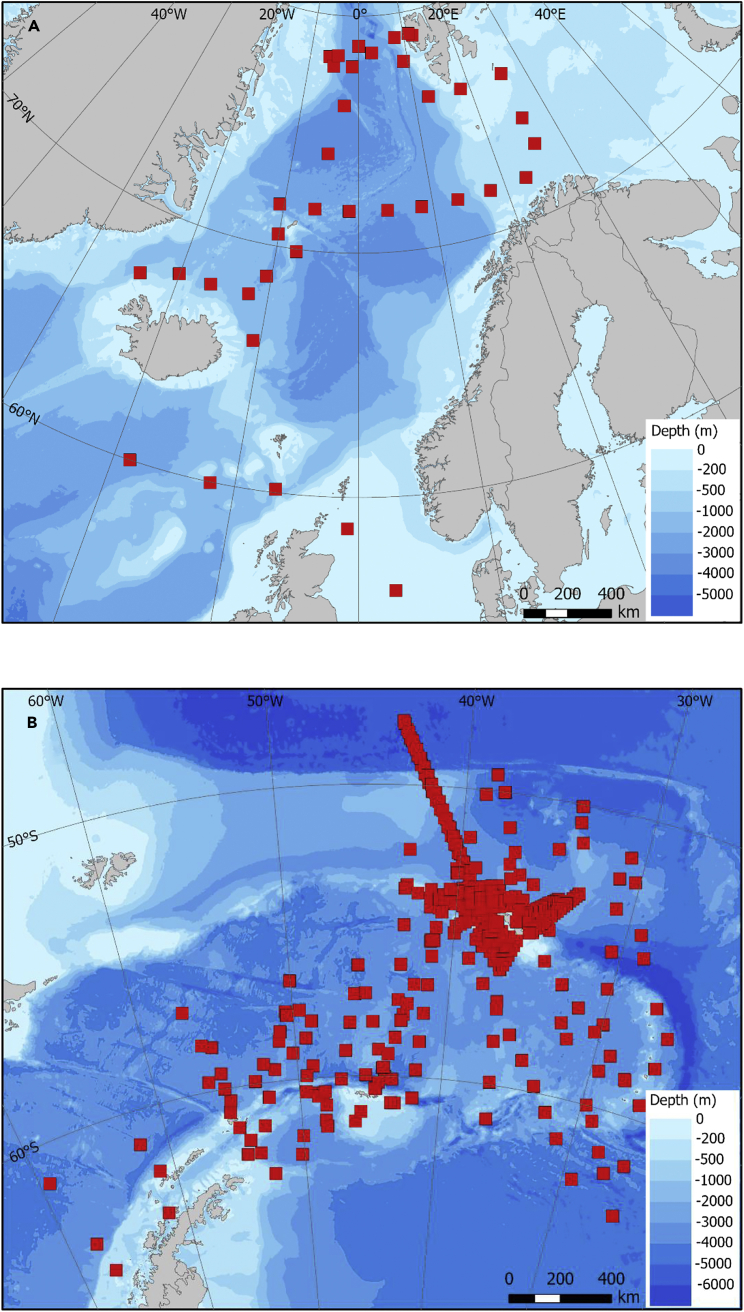
Sampling station positions Distribution of sampling stations in (A) the sub-Arctic and (B) the Southern Ocean.

**Figure 3:**
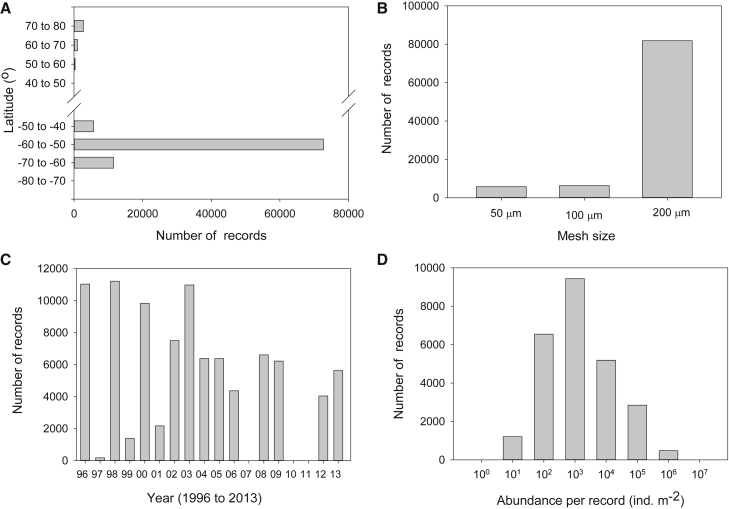
Descriptors of the database (A–D) (A) The number of records relative to latitude, (B) the number of records relative to the mesh size used, (C) the number of records taken in each year, and (D) a size frequency of abundance per record. A “record” represents each species or higher taxa identified within an individual sampling deployment.

The database comprises data from a total of 15 different oceanographic expeditions, between 1996 and 2013. All of the expeditions were carried out in the Southern Ocean with the exception of a single sub-Arctic expedition in 2012. In the sub-Arctic, the majority of records were obtained between 70° and 80°N (Figure 2A), while in the Southern Ocean it was between 50° and 60°S , with a major concentration around the island of South Georgia (Figure 2B). This region is a particularly productive part of the Southern Ocean where intense phytoplankton blooms occur, linked to the ready availability of the essential micronutrient iron and which in turn allows development of a high standing stock of zooplankton.^7^ It has been a focus of research since the 1920s, due to the location of a shore-based whaling industry and subsequently a commercial krill fishery. Across all expeditions, the most commonly deployed mesh size was 200-μm, totalling 517 separate deployments (Table 1), with the 50-μm net being deployed the least (34 times). Most of the deployments went to a maximum depth of 200 m (398 times) or 400 m (241 times), with shallower maximum depths accounting for less than 10% of total deployments. Of the 93,914 individual records of separate taxa or developmental stages across all deployments (Figure 3A), the majority were from the 200-μm mesh net deployed to a maximum of 200 m, which reflects the concentration of sampling effort within this category (Figure 3B; Table 2). A total of 295 separate taxa, developmental stages, and more general categories was recorded across all net deployments. Phylogenetically the crustacean groups Copepoda (61%) and Euphausiacea (krill) (15%) accounted for the greater majority of categories in the database, with all other groups accounting for ≤3% each.

**Table 1:** Matrix relating mesh sizes to maximum sampling depths for numbers of sampling events

Depth (m)	50	100	100–200	200	400	Grand total
Total no. of deployments						

50 μm					34	34
100 μm				48	103	151
200 μm	3	39	21	350	104	517
Grand total	3	39	21	398	241	702

A “deployment” represents every time a Bongo net was successfully sampled, noting that a number of deployments may be made per sampling station (Figure 2).

**Table 2:** Matrix relating mesh sizes to maximum sampling depths for numbers of records

Depth (m)	50	100	100–200	200	400	Grand total
Total no. of records						

50 μm					5,756	5,756
100 μm				294	6,045	6,339
200 μm	303	6,714	3,546	55,959	15,297	81,819
Grand total	303	6,714	3,546	56,253	27,098	93,914

A “record” represents each species or higher taxa identified within an individual deployment.

The number of data records varied between years with the highest number of records in 1996, 1998, and 2003 (Figure 3C). There were no records in 2007, 2010, and 2011. Individual abundance values (ind. m^−2^) of between 100 and 10,000 were the most frequently observed across the dataset (Figure 3D). Maximum values did not exceed 10,000,000 ind. m^−2^.

While analysis of the cruises’ CTD data is outside of the scope for this manuscript, processed CTD data are available from the British Oceanographic Data Centre (BODC)^8^ for 10 cruises (JR11, JR17, JR28, JR57, JR70, JR161, JR177, JR200, JR271, and JR274). These data can be either requested from the BODC help desk or from the BODC CTD profiles portal.^9^ Processed CTD data for five cruises (JR38, JR47, JR82, JR100, and JR116) are not available from the NERC Data Centres, likely because they were not submitted by the data originators.

## Discussion

The bongo net system, rather than other commonly used plankton nets, was chosen as it enabled the relatively rapid collection (∼20 min per haul) of mesozooplankton that we required for station characterization, as well as allowing the use of two mesh sizes in a single deployment. Different mesh sizes selectively capture different size fractions of plankton. The 200-μm mesh is widely used in marine research and is at the lower end of the mesozooplankton size range (0.2–2.0 cm), whereas the 100- and 53-μm meshes allowed an assessment of the smaller species and stages that basically comprise the microzooplankton (20–100 μm). We were therefore able to investigate the relative abundance and biomass retained by each across a number of cruises where results indicated that a 200-μm net captured on average 17% of the mean abundances captured by the 53-μm net and the 100-μm net 58% of the 53-μm net.^10^

The greater majority of the net deployments were to 200 m. This depth was chosen as this is the recognized extent of the oceanic epipelagic layer where most photosynthesis takes place and in summer, when the majority of cruises were undertaken, contains the majority of the mesozooplankton in the water column.

### Constraints and assumptions

Several assumptions have been made about net performance. Firstly, in the absence of flow meters, the assumption of 100% filtration efficiency is questionable, particularly where dense phytoplankton blooms are present. It has been suggested that, to achieve optimal filtration, the ratio (*w*) of open mesh area to mouth area should be around 6.^11^ Our calculations for the 200-μm Bongo net suggest that this condition is broadly met with a *w* calculated at ∼5, although for the 100-μm mesh *w* is around ∼3.5. Equations formulated by Smith et al.^12^ further suggested that net efficiency would vary according to the amount of particulate material in the water, the mesh size and the open area, and the form of the net, conical being best. Applied to our Bongo nets, these equations suggest that for “blue” oceanic water, a *w* of ∼2.3 would be sufficient, whereas in regions where particulate loading is high, a value of ∼5 would be necessary. The 200-μm mesh net meets these criteria, although the 100-μm net is likely to under-sample. The mini-Bongo, with a *w* of ∼8, appears to more than meet these criteria.

Vertically hauled nets are selective for particular size classes and likely to be avoided by highly mobile species, such as large euphausiid species, including Antarctic krill (*Euphausia superba*). Increasing the hauling speed does not necessarily reduce this issue since it generates a bow wave ahead of the net which decreases filtering and capture efficiency. Nevertheless, the capture efficiency of less mobile species is likely to be high over the short towing distances and slow hauling speeds that were employed. During a seasonal series of cruises in the Scotia Sea, a comparison of median densities of plankton species stages big enough to be retained by all three mesh sizes (200, 100, and 53-μm) indicated no significant differences in standardized abundance (ind. m^−2^) through a 400-m water column. When data from the 100- and 200-μm mesh nets were used independently to describe community structure, both indicated a similar division of species across the Scotia Sea.^10^

That phytoplankton could sometimes have influenced filtration efficiency was suggested in a study by Ward et al.,^13^ who compared the relative zooplankton abundance ratio collected by the 200-μm Bongo net relative to an N70V ring net (used by *Discovery Investigations* in the Southern Ocean during the 1920s and 1930s) when fished vertically through the same 200-m horizon at differing phytoplankton concentrations. The nets were broadly similar in design, with both being ∼2.8 m long and having similar mouth areas. The N70V was composed of three sizes of mesh, an upper section of ∼6 mm, a mid-section of 440-μm mesh, and a lower section of 195-μm mesh. The abundance ratio (Bongo: N70V) was investigated in relation to the chlorophyll *a* (Chl *a*) maximum in the upper 100 m of the water column. A drop from a ratio of ∼1.4 to ∼1.2 was observed with increasing Chl *a* up to ∼3 mg m^−3^. At the same time the proportion of copepods (a dominant component of the zooplankton) retained by both nets fell with increasing Chl *a* as a result of a positive relationship with appendicularians (filter-feeding larvaceans).^13^

Using a plankton splitter to create subsamples can also introduce bias, particularly if the individual organisms are not homogenously distributed within the body of the splitter. Any clumping requires a greater subsampling effort to offset this effect.^14^ We go some way to countering any clumping effects by removing larger organisms likely to influence whether smaller organisms are homogenously distributed or not and by counting both aliquots from the final split fraction. Plankton enumeration is time-consuming with a doubling of precision requiring a 4-fold effort in counting. For example, a precision of ±20% requires the analysis of 100 specimen, while a precision of ±10% requires 400 counts.^15^ Any of the ensuing analyses performed on the plankton datasets required log or double-root transformations of counts, further implying that the level of precision obtained was sufficient.

### Data use

During a series of earlier oceanographic studies in the Antarctic, the *Discovery Investigations*, carried out in the early part of the last century (1920s to late 1930s), it was recognized that plankton species were on the whole circumpolar^16^ and showed strong relationships with water temperature.^17^ Subsequently, samples collected using Bongo and other net samplers have contributed to the many investigations that have taken place across the entire Southern Ocean. Community studies, for example, have given a near consistent view of epipelagic communities that are bounded by physical transitions and discontinuities often associated with frontal zones.^18^ Despite the assumptions regarding Bongo net efficiency and the range of sampling scales, our studies have better defined plankton communities around South Georgia,^19^^,^^20^ across the Scotia Sea and beyond,^21^^,^^22^ as well as providing material to investigate zooplankton growth and production,^23^^,^^24^ diversity,^25^ and seasonal changes in population dynamics.^10^

**Figure 4:**
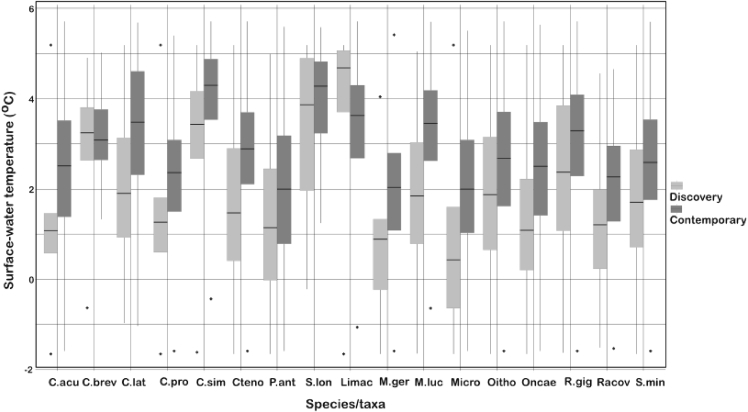
Sea surface temperature ranges of individual mesozooplankton taxa between eras Sea surface temperature ranges of 16 dominant taxa (in terms of abundance and biomass) in the Southern Ocean Atlantic sector (65–49°S, 80–20°W) during the *Discovery Investigations* (October to April 1926–1938) and contemporary times (October to April 1996–2013). The horizontal line in each box represents the median temperature of occurrence (M0). Upper and lower box limits denote the 25th and 75th percentiles, whiskers, 5th and 95th percentiles, and dots, maximum and minimum. Species listed: *Calanoides acutus* (C.acu), *Clausocalanus brevipes* (C.brev), *Clausocalanus laticeps* (C.lat), *Calanus propinquus* (C. prop), *Calanus simillimus* (C.sim), *Ctenocalanus vanus* (Cteno), *Pareuchaeta antarctica* (P.ant), *Subeucalanus longipes* (S.lon), *Metridia gerlachei* (M. ger), *Metridia lucens* (M. luc), *Microcalanus pygmaeus* (Micro), *Oithona* spp. (Oitho), *Oncaea* spp. (Oncae), *Rhincalanus gigas* (R.gig), *Racovitzanus antarctica* (Racov), *Scolecithricella minor* (S.min). Reproduced from Tarling and co-workers^28^ and republished with permission from Global Change Biology (Blackwell Publishing).

**Figure 5:**
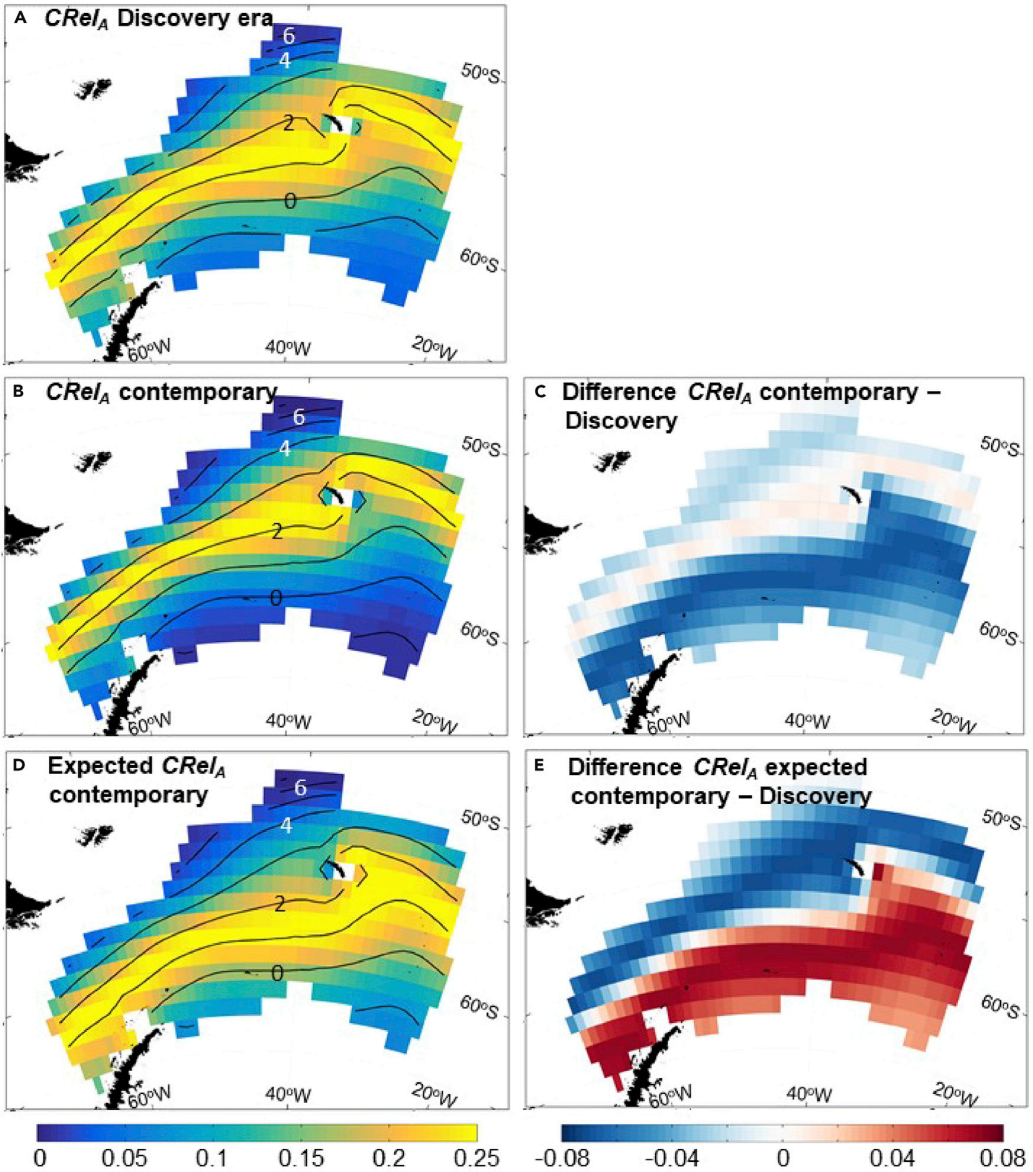
Projected distributions of observed and predicted mesozooplankton community abundance between eras Zooplankton community relative abundance anomaly (*CRelA*) as of (sea surface temperature) in the Southern Ocean Atlantic sector during the *Discovery Investigations* (October to April 1926–1938) and contemporary times (October to April 1996–2013). (A) *CRelA* for *Discovery Investigations*. (B) *CRelA* for contemporary times. (C) Difference between *CRelA* for contemporary times and *Discovery Investigations*. (D) Expected present day *CRelA* assuming the zooplankton community maintained a fixed relationship with sea surface temperature since the *Discovery Investigations*. (E) Expected difference in *CRelA* from *Discovery Investigations* era to contemporary times had the relationship to sea surface temperature remain fixed (d–a). Mean sea surface isotherms (°C) for October–April for the *Discovery Investigations* (A) and contemporary times (B and D) are plotted. The derivation of *CRelA* and how it is projected in these figures is explained further in the supplemental information. The figure is reproduced from Tarling and co-workers^28^ and republished with permission from Global Change Biology (Blackwell Publishing).

The importance of understanding the distribution and composition of plankton, its relationship to ocean physics and its linkages in the food chain is particularly acute at a time when the oceans generally are warming.^26^ Plankton by virtue of their relatively short life cycles, linkages to ocean currents, and, in many cases, sensitivity to environmental temperature are seen by many as sentinels of climate change.^27^ An understanding of species distributions and community structure will provide a benchmark against which future change can be assessed. To this end, comparisons of the distribution of plankton captured during the *Discovery Investigations* in the 1920–1930s and from Bongo net samples in contemporary times were undertaken.^28^ It was found that in the ∼75 years that had elapsed since the 1920s, the relationship with temperature had changed, with the temperature of community peak abundance being some 0.98°C warmer in contemporary times. The sea surface temperature ranges of 16 dominant copepod species for each era are shown in Figure 4. Almost without exception, species temperature ranges were warmer than they were last century by ∼0.75°C. However, looked at in spatial terms, and at odds with the hypothesis that plankton would conserve their thermal niche by moving south as waters warm, their geographic distributions remained much the same (Figure 5). These findings suggest that factors other than temperature, such as food availability and life cycle patterns, may be significant in promoting levels of resilience to climatic change among Southern Ocean mesozooplankton.

Efforts to understand these complexities and to parameterize resilience in a mechanistic way will be increasingly important in the years ahead and thus the storage of such baseline data in repositories, such as the UK Polar Data,^29^ and its ease of access and availability to the wider community is paramount.

## Experimental procedures

### Resource availability

#### Lead contact

Further information and requests for resources should be directed to and will be fulfilled by the lead contact, Petra ten Hoopen (peopen@bas.ac.uk).

#### Materials availability

Samples generated in this study have been deposited to the British Antarctic Survey Sample Stores and can be made available upon request.

### Net sampling and analytical methodologies

The size of the mesh used in any net is generally determined in relation to the size of plankton targeted. Plankton by definition are generally drifting organisms dispersed by ocean currents and it is convenient to group them into the following categories; macrozooplankton (>2–20 cm), including euphausiids, amphipods, and smaller jellyfish, mesozooplankton (0.2–2.0 cm), including copepods and ostracods, as well as pteropod molluscs and chaetognaths and microzooplankton (20–200_μm), such as large protozoans, copepod nauplii, foraminiferans, and tintinnids. The main focus of the majority of the Bongo net hauls was the mesozooplankton taken using a 200-μm mesh and, to a lesser extent, the microzooplankton more adequately sampled using the 100-μm net and the mini-Bongo with a 53-μm mesh. The mesozooplankton includes the crustacean group Copepoda, which generally dominates in terms of biomass and grazing activity within this size group and typically comprises >75% of plankton biomass.

The motion-compensated Bongo net was designed to capture zooplankton in good condition both for characterization and enumeration as well as ensuring that live undamaged material could be obtained for experimental use. Two nets, 2.8 m long and 0.61 m diameter with solid cod-ends, were attached side by side and a towing wire connected to a motion-compensating mechanism within the net frame, operated through coiled springs that damp movement imparted by rolling of the ship. The nets, one with a mesh size of 100 μm and the other with a mesh size of 200-μm, were deployed from the midships gantry of RRS *James Clark Ross* with the ship stationary and head to wind. They were lowered vertically to the required depth (usually 200 m) where water depth permitted, or within 10 m of bottom depth (as determined from the ship’s echo sounder) when the sea was shallower, and then hauled vertically to the surface at ∼0.22 m s^−1^. However, during four cruises, samples were collected between 400 and 0 m because investigators were interested in collecting species that had recently over-wintered at depth and were estimated to lie largely below 200 m.

Flow meters were not used to record volume swept by the nets as the slow hauling speed was at the bottom end of the calibration range of most flow meters available. Instead, it was assumed that the filtration was 100% efficient and volume swept was determined by calculating the mouth area and multiplying by the vertical sampling interval. For a 200-m water column, this represented ∼58 m^3^. During some cruises, a mini-Bongo was also deployed. This was 2.3 m long with a mouth diameter of 0.18 m and was equipped with 53-μm mesh nets. For a water column of 200 m, this net swept ∼5 m^3^. This net was not equipped with a motion-compensation mechanism.

Sampled plankton were transferred from each net’s cod-end into separate buckets part-filled with seawater at ambient temperature and taken into the laboratory where they were concentrated by gentle filtration through a filter of the same mesh size as the net. Each sample was then placed in fixative (10% v/v seawater formaldehyde, equivalent to 4% w/v) and transferred to a storage jar along with a label detailing pertinent information, such as date, cruise, net type and mesh size, and event and station number. Occasionally samples were too large to fit comfortably into the largest preserving jar, in which case they were suspended in a known volume of seawater, mixed, and an aliquot taken by decanting part of the suspended sample into a measuring beaker. We tried to ensure that the ratio of fixative to sample was ∼1:10. Whether the sample was entire or a known aliquot was also indicated on the sample label.

Following transfer to the home laboratory, plankton samples were drained of formaldehyde, gently rinsed in freshwater, and placed in Steedman’s solution, a preservative consisting of propylene glycol, propylene phenoxetol, buffered formaldehyde, and deionized water.^30^ Preserved samples were initially systematically examined for large macroplankton, such as krill or salps, which were removed before the residue was placed in a two-chambered Folsom plankton splitter and serially split through repeated halving until it was estimated that a representative and countable set of aliquots had been reached. Initially the sample was split into two halves and then either the right (R) or left (L) half would be further split, and so on alternately, i.e., 1/2R, 1/4L, 1/8R, 1/16L *et seq*. When a split level had been achieved such that an aliquot was estimated to contain the desired number of organisms, both the left and right halves were counted.^31^ Larger mesozooplankton were usually counted from split fractions ranging from 1/16 to 1/64, whereas smaller species and stages were counted from smaller fractions as they are always more abundant in samples and hence further splitting was required. Following this, split samples were examined under a Nikon SMZ 10 binocular microscope. The aim was, where possible, to count between 500 and 1,500 species stages from each sample, the higher number of individuals generally being enumerated in the largest samples. We assumed that animals were randomly distributed during the splitting procedure, giving a 0.95 confidence interval of between ±10% and ±5%^15^ Numbers were standardized to individuals m^−2^ through dividing by the estimated volume filtered (m^3^) and multiplying by maximum sampling depth (m).

## Data Availability

• Biological data from Bongo plankton samples have been deposited at the UK Polar Data Centre under https://doi.org/10.5285/5A711904-EF42-46A3-9F47-3F0D6B231F65 and are publicly available as of the date of publication. • This paper does not report original code. • Any additional information required to reanalyze the data reported in this paper is available from the lead contact upon request.

## Supplemental information

Document S1. Experimental procedures

Document S2. Article plus supplemental information

## References

[1] Fraser J.H. (1968). Zooplankton Sampling.

[2] Keynes R., Darwin C. (2001). Charles Darwin's zoology notes & specimen lists from HMS Beagle. J. Hist. Biol..

[3] Wiebe P.H., Benfield M.C. (2003). From the Hensen net toward four-dimensional biological oceanography. Prog. Oceanogr..

[4] McGowan J.,A., Brown D.,M. (1966).

[5] ten Hoopen P., Peat H.J., Ward P., Tarling G.A. (2022). Polar biodiversity data: From a national marine platform to a global data portal. Patterns.

[6] Ward P., Tarling G., Shreeve R., ten Hoopen P. (2020).

[7] Atkinson A., Whitehouse M.J., Priddle J., Cripps G.C., Ward P., Brandon M.A. (2001). South Georgia, Antarctica: a productive, cold water, pelagic ecosystem. Mar. Ecol. Prog. Ser..

[8] The British Oceanographic Data Centre (2022). https://www.bodc.ac.uk/.

[9] The British oceanographic data Centre CTD profiles portal. https://www.bodc.ac.uk/data/bodc_database/ctd/search/.

[10] Ward P., Atkinson A., Tarling G. (2012). Mesozooplankton community structure and variability in the Scotia Sea: a seasonal comparison. Deep Sea Res. Part II: Topical Studies in Oceanography.

[11] Tranter D.,J., Smith P.,E. (1968). Filtration performance. UNESCO Monogr. Oceanogr. Methodol..

[12] Smith P.E., Counts R.C., Clutter R.,I. (1968). Changes in filtering efficiency of plankton nets due to clogging under tow. ICES J. Mar. Sci..

[13] Ward P., Tarling G.A., Coombs S.H., Enderlein P. (2012). Comparing Bongo net and N70 mesozooplankton catches: using a reconstruction of an original net to quantify historical plankton catch data. Polar Biol..

[14] Griffiths F.B., Brown G.H., Reid D.D., Parker R.R. (1984). Estimation of sample zooplankton abundance from Folsom splitter sub-samples. J. Plankton Res..

[15] Lund J.W.G., Kipling C., Le Cren E.D. (1958). The inverted microscope method of estimating algal numbers and the statistical basis of estimations by counting. Hydrobiol. (Sofia).

[16] Baker A. de C. (1954). The circumpolar continuity of Antarctic plankton species. Discov. Rep..

[17] Mackintosh N.,A. (1936). Distribution of the macroplankton in the Atlantic sector of the Antarctic. Discov. Rep..

[18] Boltovskoy D., Gibbons M.J., Hutchings L., Binet D., Boltovskoy D. (1999). South Atlantic Zooplankton 1.

[19] Ward P., Shreeve R., Whitehouse M., Korb B., Atkinson A., Meredith M., Pond D., Watkins J., Goss C., Cunningham N. (2005). Phyto-and zooplankton community structure and production around South Georgia (Southern Ocean) during Summer 2001/02. Deep Sea Res. Oceanogr. Res. Pap..

[20] Ward P., Whitehouse M., Shreeve R., Thorpe S., Atkinson A., Korb R., Pond D., Young E. (2007). Plankton community structure south and west of South Georgia (Southern Ocean): links with production and physical forcing. Deep Sea Res. Oceanogr. Res. Pap..

[21] Ward P., Shreeve R., Atkinson A., Korb B., Whitehouse M., Thorpe S., Pond D., Cunningham N. (2006). Plankton community structure and variability in the Scotia Sea: austral summer 2003. Mar. Ecol. Prog. Ser..

[22] Ward P., Whitehouse M., Brandon M., Shreeve R., Woodd-Walker R. (2003). Mesozooplankton community structure across the antarctic circumpolar current to the north of South Georgia: Southern Ocean. Mar. Biol..

[23] Shreeve R.S., Ward P., Whitehouse M.J. (2002). Copepod growth and development around South Georgia: relationships with temperature, food and krill. Mar. Ecol.: Prog. Ser..

[24] Shreeve R.S., Tarling G.A., Atkinson A., Ward P., Goss C., Watkins J. (2005). Relative production of *Calanoides acutus* (Copepoda: calanoida) and *Euphausia superba* (Antarctic krill) at South Georgia, and its implications at wider scales. Mar. Ecol. Prog. Ser..

[25] Woodd-Walker R.S., Ward P., Clarke A. (2002). Large-scale patterns in diversity and community structure of surface water copepods from the Atlantic Ocean. Mar. Ecol. Prog. Ser..

[26] Beaugrand G., Edwards M., Brander K., Luczak C., Ibanez F. (2008). Causes and projections of abrupt climate-driven ecosystem shifts in the North Atlantic. Ecol. Lett..

[27] Richardson A.J. (2008). In hot water: zooplankton and climate change. ICES J. Mar. Sci..

[28] Tarling G.A., Ward P., Thorpe S.E. (2018). Spatial distributions of Southern Ocean mesozooplankton communities have been resilient to long-term surface warming. Global Change Biol..

[29] The UK Polar Data Centre (2022). https://www.bas.ac.uk/data/uk-pdc/.

[30] Steedman H.R. (1976). Zooplankton fixation and preservation. UNESCO Monogr. Oceanogr. Methodol..

[31] McEwen G.F., Johnson M.W., Folsom T.R. (1954). A statistical analysis of the performance of the Folsom plankton sample splitter, based upon test observations. Archiv für Meteorologie, Geophysik und Bioklimatologie, Serie A.

