# Supplementary material for: A database of zooplankton abundance in the Atlantic sectors of the Southern and sub-Arctic Oceans
Source: Patterns (N Y). 2022 Aug 30;3(10):100554. doi: 10.1016/j.patter.2022.100554 (PMC9583039; doi:10.1016/j.patter.2022.100554)
Supplement: Document S1. Experimental procedures [file mmc1.pdf]

Patterns, Volume *3*

Supplemental information

A database of zooplankton abundance in the Atlantic sectors of the Southern and sub-Arctic Oceans

Peter Ward, Geraint A. Tarling, and Petra ten Hoopen

Supplemental experimental procedures

The following provides supplementary information to support the derivation of the datasets illustrated in Figure 5, as originally published in Tarling et al.[1]. In particular, the information explains how the metric $CRel_A$ is derived, which is the principal parameter plotted in Figure 5.

Mesozooplankton species composition and abundance

The data was obtained by net sample analyses from 155 stations south of the Polar Front in the southwest Atlantic sector of the Southern Ocean, collected as part of the *Discovery Investigations* (1926-1938). The samples were obtained via a series of N70V plankton nets deployed vertically between 250 m and the surface, following Kemp et al.[2]. Mesh sizes in this net decrease in stages from 5 mm in the upper part, to 440 µm and then 195 µm in the mid and lower parts respectively (measurements are metric equivalents of the original imperial units). A further 451 samples were analysed from nets taken in the same ocean sector in contemporary times (1996-2013). The contemporary samples were obtained from deployments of either RMT1 nets (330 µm mesh) fished obliquely from 200-0 m or Bongo nets (200 µm) fished vertically from either 200-0 m or 400-0 m. During sample analysis, larger organisms were either enumerated from complete samples, or the whole sample placed in a Folsom plankton splitter and fractionated into replicate aliquots until countable numbers (~200 individuals) were estimated to be present. For smaller, more numerous organisms, the sample was further fractionated until countable replicates (~500-800 organisms) were obtained. Abundances of taxa were standardised according to the split fraction, and the amount of water each net filtered was estimated based on mouth area and distance towed, to derive individual species concentrations (ind. m^{-3}).

Sea surface temperature

Sea surface temperature was measured at the zooplankton sampling sites and additional locations using water bottle samples during the *Discovery Investigations*, following Kemp et al.[2] and with high resolution conductivity temperature depth (CTD) instruments during the contemporary cruises, following Whitehouse et al.[3].

To determine the change in sea surface temperature between the 1920s-1930s and present day, data from all available stations from the *Discovery Investigations* and contemporary cruises between the months of October and April and located south of the Polar Front in water depths >500 m were analysed for the region 65-49°S, 80-20°W. Stations were identified as being south of the Polar Front from their vertical temperature profile, see Gordon et al.[4]. Duplicate temperature measurements, defined as measurements taken within 1 day and 5 km of another sample, were excluded. The surface temperature datasets were compared with the World Ocean Atlas (WOA) 2013 v2 monthly mean 1° surface climatology for 2005-2012, following Locarnini et al.[5]. For each dataset, nearest neighbour interpolation was used to extract the WOA temperature at each station from the corresponding mean monthly objectively analysed field at 0 m depth. WOA grid cells with zero contributing data points were excluded, see Meredith and King[6]. The resulting differences between the station data points and the WOA temperature fields were averaged at 1° spatial resolution to remove geographic bias from the station coverage. The median offset from the WOA data for the gridded data from the *Discovery Investigations* and contemporary data was -0.78°C and -0.04°C, respectively (N = 106 for Discovery, N = 91 for contemporary).

We used these offsets to produce mean October-April sea surface temperature fields for the *Discovery Investigations* and contemporary eras. WOA monthly mean 2005-2012 climatological temperature fields at 0 m for October to April were averaged and the corresponding offset applied uniformly. In this case, all grid cell values were retained to provide a spatially complete coverage for the study region.

Numerical methods

Mesozooplankton data analyses focussed on the crustacean fraction which made up approximately 90% of all organisms within net samples. Analyses considered 16 taxa from this fraction chosen because they were major contributors to either the abundance or the biomass of the samples (biomass was determined through multiplying abundance by typical dry weight and was calculated only for the purpose of identifying species that were less abundant but still major contributors through

their large individual size). Analyses were restricted to net samples taken between October and April. The datasets were normalised such that each taxon made an equal contribution to the metric of community response (*CRel*, see below) and that the influence of less abundant species was the same as more abundant ones. This ensured that the response metric was not dominated by a small number of highly abundant taxa.

The first stage in this process was to derive relative abundance for each taxon, as follows:

$$Rel_{x,y} = \frac{Abs_{x,y}}{\sum_{y=1}^{y=n} Abs_x} \quad (1)$$

where *Rel* is relative abundance, *Abs*, absolute abundance (ind. m^{-3}), *x,* taxon, *y,* the sample station and *n*, the total number of sampling stations. For the contemporary sample set, it was necessary to determine *Rel* separately for three different types of net deployment (RMT1 200-0 m, Bongo 200-0 m and Bongo 400-0 m). The resulting three matrices were subsequently concatenated into a single matrix before further analysis. Community relative abundance (*CRel*) was calculated as:

$$CRel = \sum_{x=1}^{x=16} Rel_y \quad (2)$$

Cumulative probability curves were generated for (i) cumulative station rank as a function of sea surface temperature and (ii) cumulative *Rel* or *CRel* as a function of sea surface temperature. Medians and percentiles of *Rel* were calculated, from which the corresponding temperatures were determined to ascertain the relationship of each taxon to temperature. For *CRel*, the residual difference between (i) and (ii), the community relative abundance anomaly ($CRel_A$), was calculated to derive a metric of abundance that normalises for the distribution of sample stations between sea surface temperatures. $CRel_A$ trajectories were fitted by a Gaussian distribution function ($G_{(T°C)}$) where the inflection point, X_0, denotes the sea surface temperature at which peak relative abundance occurs. The respective $G_{(T°C)}$ functions were plotted spatially, using the sea surface temperature fields described above, to identify regions of peak $CRel_A$. A bootstrapping analysis was performed to determine the level of difference in X_0 between the *Discovery Investigations* and contemporary datasets, following Hilborn and Mangel[7]. $CRel_A$ was resampled through selecting 148 datapoints with replacement from either the *Discovery Investigations* or contemporary datasets 30 times. $G_{(T°C)}$ and the value of X_0 was derived for each resampled dataset. The significance level of the difference in X_0 between *Discovery Investigations* and contemporary datasets was tested by a two tailed t-test, having first passed tests for Normality (Shapiro-Wilk) and Equal Variance. It was not possible to fit a Gaussian distribution to the temperature distributions of a number of individual taxa since they were not normally distributed, so the median temperature of occurrence (termed M_0) was derived for all individual taxa for comparative purposes.

Comparison of relative abundance ranks between eras

To establish whether community structure had altered between eras, rankings in relative abundance of individual taxa were compared. For contemporary samples, only the Bongo nets that fished from 200-0 m were used for the comparison, given that they were the closest in sampling method to the N70V nets. Differences in the sampling efficiencies of the nets were accommodated through conversion factors derived from corresponding *in situ* net trials, following Ward et al.[8]. Briefly, when both nets were fished to a depth of 200 m, the Bongo net captured ~3 times more copepods overall than the N70V and ~4 times more if the <0.5 mm body length size class alone was considered. Above a body length of 1 mm, the difference in favour of the Bongo net was broadly invariant, averaging 1.7 across all other classes. Therefore in the case of copepod life stages in the <0.5 mm and 0.5–0.99 mm size classes, which numerically dominated the plankton, we determined the Bongo: N70V ratio for each individual taxon and applied this factor to the N70V data. For size groups >1 mm, the average factor of 1.7 was applied. Where stage structure of a species was not distinguished we summed abundances of all stages and used an average factor. In the present study, the N70V nets routinely divided the 250-0 m water column into 3 depth strata (250-100 m, 100-50 m and 50-0 m) and so, for each station, the contents of each net were summed and averaged over the entire 250 m.

As the Bongo nets only fished from 200-0 m, we multiplied the N70V catch data by 250/200 to provide a conservative m^{-3} abundance estimate to compensate for the difference in depth.

Projections

Geographic projections of $CRel_A$ show that community peak abundance occurred in much the same locations in both eras (Figure 5), which was around 60°S to 62°S in the Drake Passage, then broadening and moving northwards across the Scotia Sea to envelope South Georgia, before narrowing into a band between 52°S and 55°S east of South Georgia. Comparatively, the distribution of peak abundance was slightly wider in 1920s-1930s (Figure 5a) than in contemporary times (Figure 5b), which reflects the broader temperature relationship function in the former era, see Tarling et al. Supplementary Information[1]. In Figure 5d, we assume that the mesozooplankton community maintains its relationship to surface temperature as observed in the 1920s-1930s and project this relationship onto the surface temperature conditions of the contemporary era. This predicts that peak community abundance would occur further south by approximately 500 km, occurring below 62°S in Drake Passage and remaining south of South Georgia and below 55°S further east. This projection severely underpredicts mesozooplankton community abundance levels in the northern half of the survey region and overpredicts it to the south, compared to contemporary observations (Figure 5e).

Supplemental References

1. Tarling, G.A., Ward, P., and Thorpe, S.E. (2018) Spatial distributions of Southern Ocean mesozooplankton communities have been resilient to long-term surface warming. Global Change Biology 24, 132-42. doi:10.1111/gcb.13834.

2. Kemp, S., Hardy, A.C., and Mackintosh, N.A. (1929). Discovery investigations: Objects, equipment and methods. Disc. Reps 1, 141-232.

3. Whitehouse, M.J., Meredith, M.P., Rothery, P., Atkinson, A., Ward, P. and Korb, R.E. (2008). Rapid warming of the ocean around South Georgia, Southern Ocean, during the 20th century: Forcings, characteristics and implications for lower trophic levels. Deep-Sea Res. Part I: Oceanographic Research Papers 55, 1218-1228. doi:10.1016/j.dsr.2008.06.002.

4. Gordon, A.L., Georgi, D.T., and Taylor, H.W. (1977). Antarctic polar frontal zone in the western Scotia Sea - summer 1975. J. Phys. Oceanog. 7, 309-328.

5. Locarnini, R.A., Mishonov, A.V., Antonov, J.I., Boyer, T.P., Garcia, H.E., Baranova, O.K., Zweng, M.M., Paver, C.R., Reagan, J.R., Johnson, D.R., Hamilton, M., and Seidov, D. (2013) World Ocean Atlas 2013, Volume 1: Temperature. In *NOAA Atlas NESDIS 73,* Levitus, S., and Mishonov, E.A., ed., pp. 40.

6. Meredith, M.P., and King, J.C. (2005). Rapid climate change in the ocean west of the Antarctic Peninsula during the second half of the 20th century. Geophys. Res. Lett., L19604, doi:10.1029/2005GL024042.

7. Hilborn, R., and Mangel, M. (1997) .*The ecological detective,* Princeton, Princeton Univ. Press.

8. Ward, P., Tarling, G. A., Coombs, S. H., and Enderlein, P. (2012). Comparing Bongo net and N70 mesozooplankton catches: using a reconstruction of an original net to quantify historical plankton catch data. Polar Biol. *35*, 1179-1186. https://doi.org/10.1007/s00300-012-1163-x
